# Natural Xanthine Oxidase Inhibitor 5-*O*-Caffeoylshikimic Acid Ameliorates Kidney Injury Caused by Hyperuricemia in Mice

**DOI:** 10.3390/molecules26237307

**Published:** 2021-12-01

**Authors:** Dong Zhang, Mojiao Zhao, Yumei Li, Dafang Zhang, Yong Yang, Lijing Li

**Affiliations:** 1 College of Pharmacy, Changchun University of Chinese Medicine, Boshuo Road 1035, Changchun 130117, China; zhangdong_1202@126.com (D.Z.); liyumei_10@163.com (Y.L.); 2 Department of Chinese Medicine and Health Care, Changchun Humanities and Sciences College, Boshuo Road 1488, Changchun 130117, China; mojiao_zhao@163.com (M.Z.); zdf0431@126.com (D.Z.); 3 School of Pharmaceutical Sciences, Jilin University, Fujin Road 1266, Changchun 130021, China

**Keywords:** *Smilacis Glabrae Rhizoma*, 5-*O*-caffeoylshikimic acid, potassium oxonate, hyperuricemia, XOD inhibitor

## Abstract

Xanthine oxidase (XOD) inhibition has long been considered an effective anti-hyperuricemia strategy. To identify effective natural XOD inhibitors with little side effects, we performed a XOD inhibitory assay-coupled isolation of compounds from *Smilacis Glabrae Rhizoma* (SGR), a traditional Chinese medicine frequently prescribed as anti-hyperuricemia agent for centuries. Through the in vitro XOD inhibitory assay, we obtained a novel XOD inhibitor, 5-*O*-caffeoylshikimic acid (#**1**, 5OCSA) with IC_50_ of 13.96 μM, as well as two known XOD inhibitors, quercetin (#**3**) and astilbin (#**6**). Meanwhile, we performed in silico molecular docking and found 5OCSA could interact with the active sites of XOD (PDB ID: 3NVY) with a binding energy of −8.6 kcal/mol, suggesting 5OCSA inhibits XOD by binding with its active site. To evaluate the in vivo effects on XOD, we generated a hyperuricemia mice model by intraperitoneal injection of potassium oxonate (300 mg/kg) and oral gavage of hypoxanthine (500 mg/kg) for 7 days. 5OCSA could inhibit both hepatic and serum XOD in vivo, together with an improvement of histological and multiple serological parameters in kidney injury and HUA. Collectively, our results suggested that 5OCSA may be developed into a safe and effective XOD inhibitor based on in vitro, in silico and in vivo evidence.

## 1. Introduction

Hyperuricemia (HUA) is caused by disorder of purine metabolism, with abnormal elevation of serum uric acid (sUA) as hallmarks (male ≥ 420 μM or female ≥ 360 μM) [1]. Gout is a typical inflammatory arthritis induced by HUA with strong feeling of pain and has been first described as “feeling of tiger bites” in *Danxi xinfa* by Danxi Zhu (1347, Yuan Dynasty) [2]. Moreover, HUA could also result in other comorbidities, including cardiovascular diseases [3], obesity [4], diabetes [5], hypertension [6], kidney injury [7] and so on. Risk factors for development of HUA include genetics [8] (male gender, SLC2A9, ABCG2, SLC17A1/SLC17A3 and so on), dietary [9,10] (seafood, red meat, beer, spirits and so on), drugs [11] (diuretics, cyclosporin, tacrolimus, angiotensin-converting-enzyme inhibitors, non-losartan angiotensin II receptor blockers, β blockers, pyrazinamide, ritonavir and so on) and others [12]. Nowadays, 17.4% of general population of China, ranging from 15.5 to 24.6% by regions, suffered from HUA[13]. Henceforth, HUA has already become the “fourth highest” disease after hypertension, hyperlipidemia and hyperglycemia [14]. The development of sUA-lowering drugs has clear clinical significance for HUA and related diseases [15].

Abnormal elevation of sUA is mainly caused by over-production or reduced excretion of UA [16]. Xanthine oxidase (XOD) is the key enzyme for UA production, which catalyzed oxidation of hypoxantine (HX) to xanthine and further into UA together with reactive oxygen species (ROS) [17]. XOD inhibitors have long been used as principle agent utilized for reducing elevated uric acid levels in HUA or gout treatment, including purine-like inhibitors (allopurinol (AP) approved in 1966 [18]) and non-purine inhibitors (febuxostat approved in 2009 [19] and topirastat approved in 2013 [20]). Although the existing XOD inhibitor drugs have exhibited notably sUA-lowering capacity, their side effects including severe kidney dysfunction, liver impairment, gastrointestinal irritation, bone marrow suppression and even secondary HUA limited their clinical applications [21]. To minimize the side effects of XOD inhibitors, increasing demands are required to identify XOD inhibitors from natural product with more pronounced effectiveness and safety [22].

*Smilacis Glabrae Rhizoma* (SGR), is a traditional Chinese medicine (TCM) widely used in joint pain relief, deoxidation and anti-HUA for thousands of years [23]. Several TCM formulas handed down from ancient China, including modified *Simiao* decoction [24], *Zishen Shengqi* decoction [25], *Selaginella moellendorffii* prescription [26], *Qingre Chubi* decoction and modified *Guizhi Shaoyao Zhimu* decoction [27], all containing SGR as the prime medicinal ingredient. Instead of TCM formulas with multiple drugs, Hong et al. [28] found that SGR crude drug alone could also attenuates HUA in rats probably through increasing the expression of catalase. Additionally, Liang et al. [23] found water extract of SGR could ameliorate chronic HUA and gout induced by potassium oxonate (PO) and monosodium urate (MSU) in mice. Performing HPLC-DAD-MS/MS analysis, nine compounds were identified in the extract of SGR, including 5-*O*-caffeoylshikimic acid (5OCSA), isoengeletin, taxifolin, engeletin, resveratrol, astilbin and its three stereoisomers [23]. Similarly, Xu et al. [29] also found ethanol extract of SGR showed hypouricemic effects in HUA rats. Moreover, Xu et al. revealed that concentrations of seven constituents identified in the serum of HUA rats were correlated with the hypouricemic effect, including 5-*O*-caffeoylshikimic acid glucuronide, palmitic acid, 3′-*O*-methyl taxifolin glucuronide, palmitic acid, 3′-*O*-methyl taxifolin glucuronide, 3′-*O*-methyl astilbin glucuronide, astilbin glucuronide, resveratrol glucuronide, and dihydrokaempferol [29].

Based on the above-mentioned evidence, SGR extracts could be served as a promising resource for development of natural drugs preventing HUA. However, to our knowledge, there still remains a lack of clarity about which compounds from SGR are responsible for the XOD inhibitory and hypouricemic effect. Therefore, to identify natural XOD inhibitors from SGR, we performed a XOD inhibitory assay-guided separation and verified the XOD inhibitory and hypouricemic effect in a PO/HX-induced HUA mice model.

## 2. Results

### 2.1. Bioassay-Guided Isolation and Identification

**Figure 1:**
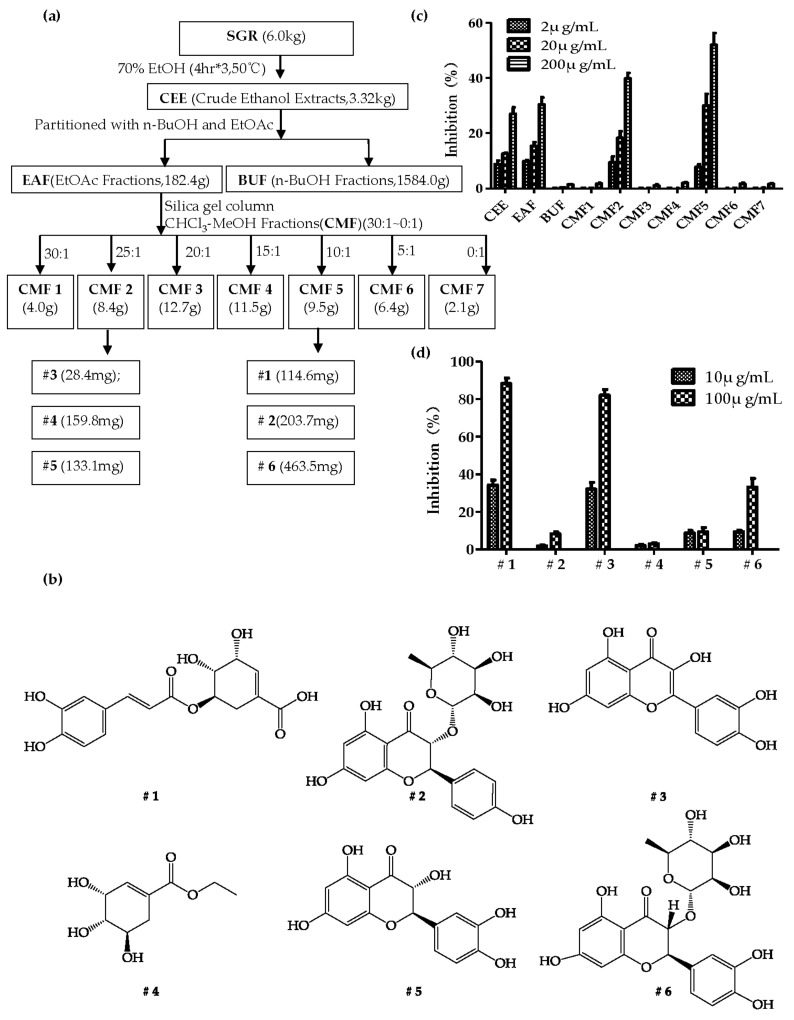
Bio-assay guided fractionation and isolation from SGR. Schematic isolation procedures of fractions and compounds from SGR were described in (**a**). Structures of the chemicals (**#1–6**) were shown in (**b**). Inhibition rates of 10 fractions (**c**) and 6 compounds (**d**) on XOD were evaluated and plotted by Graphpad. Values were presented as mean ± SEM of five replicates. SGR: *Smilacis Glabrae Rhizoma*; CEE: Crude Ethanol Extracts; EAF: EtOAc Fractions; BUF: n-BuOH Fractions; CMF: CHCl_3_-MeOH Fractions.

From the initial 6.0 kg dried SGR, we obtained 3.32 kg crude ethanol extracts (CEE) after three extractions by 70% EtOH at 50 °C (Figure 1a). Evaluated by bioassay or XOD inhibitory assay*,* we found CEE exhibited dose-dependent XOD inhibitory capacities in vitro at the concentrations of 2 μg/mL, 20 μg/mL and 200μg/mL, and reached an inhibition rate of 27.04% at 200 μg/mL (Figure 1c). After subsequent partition with *n*-BuOH and EtOAc, we obtained 182.4 g EtOAc soluble fraction (EAF) and 1584.0 g *n*-BuOH soluble fraction (BUF). Determined by bioassay, we found EAF, not BUF, showed comparable inhibitory capacity with CEE, with an inhibition rate of 30.36% in 200 μg/mL (Figure 1c). Henceforth, EAF was further subjected to silica gel column and yielded seven fractions, CMFs (CHCl_3_-MeOH fractions) 1–7, by gradient elution with CHCl_3_-MeOH (30:1–0:1 *v/v*) (Figure 1a). Evaluated by XOD inhibitory assay, CMF2 and CMF5 demonstrated a remarkable XOD inhibitory capacity, with inhibition rates of 39.82% and 52.03% at 200 μg/mL, respectively (Figure 1c).

To identify natural compounds with XOD inhibitory capacity from SGR, CMF2 and CMF5, we further isolated the compounds according to the detailed procedures described in Figure S1. Finally, we obtained compounds **3** (28.4 mg), **4** (159.8 mg) and **5** (133.1 mg) from CMF2, as well as compounds **1** (114.6 mg), **2** (203.7 mg) and **6** (463.5 mg) from CMF5. According to physicochemical properties, NMR spectral analysis (Supplementary Figure S2) and comparison with known samples [30,31,32], compounds **1**–**6** were identified as 5-*O*-caffeoylshikimic acid (**1**), engeletin (**2**), quercetin (**3**), shikimic acid ethyl ester (**4**), taxifolin (**5**) and astilbin (**6**) (Figure 1b).

### 2.2. Effects of Compounds on XOD Inhibitory Activity

**Figure 2:**
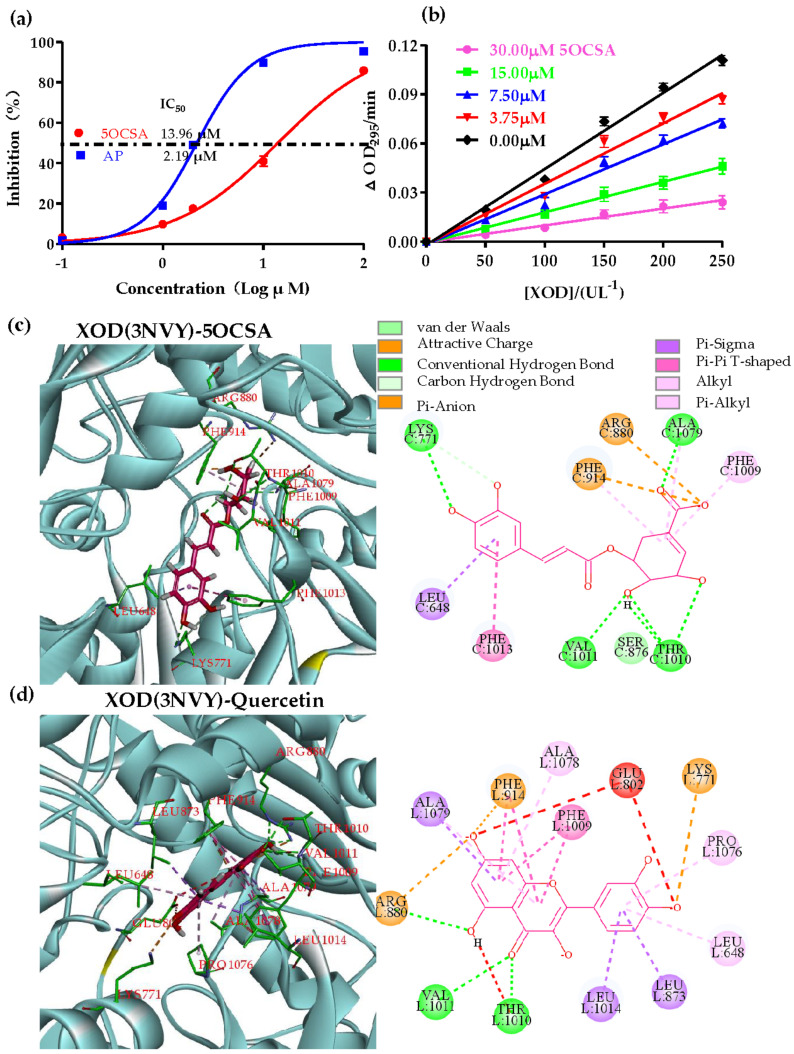
Interaction between 5OCSA and XOD in vitro and in silico. (**a**) IC_50_ of 5OCSA and AP on XOD. Plots of *v* (∆OD_295_/min)/XOD concentration with different concentrations of 5OCSA (**b**). Predicted binding mode of 5OCSA docked into XOD. 3D and 2D docking patterns of 5OCSA (**c**) and quercetin (**d**) into 3NVY has been generated by Discovery Studio 4.5. 5OCSA: 5-O-caffeoylshikimic acid; XOD: xanthine oxidase; AP: allopurinol.

To figure out the compounds responsible for the XOD inhibition in CMF2 and CMF5, we performed XOD inhibitory assays with individual compounds (**1**–**6**) at concentrations of 10 μg/mL and 100 μg/mL. Compounds **1** and **6** from CMF5 and compound **3** from CMF2 could inhibit 88.31%, 33.17% and 82.10% of XOD activity at 100 μg/mL, respectively (Figure 1d). Notably, it was the first time 5-*O*-caffeoylshikimic acid (5OCSA, #**1**) has been identified as a XOD inhibitor. To calculate the IC_50_ of 5OCSA on XOD, we evaluated the inhibition rates with different concentrations of 0.1, 1.0, 2.0, 10.0 and 100.0 μM (10.0 μg/mL 5OCSA is close to 3.0 μM). Through non-linear regression analysis, the IC_50_ value of 5OCSA was calculated as 13.96 μM (Figure 2a). AP was used as control and the IC_50_ value was 2.19 μM (Figure 2a), which is consistent with previous reports [33,34]. To determine the type of interaction between 5OCSA and XOD, we plotted OD velocity (∆OD_295_/min)/[XOD] at 0.00, 3.75, 7.50, 15.00, and 30.00 μM of 5OCSA respectively (Figure 2b). The straight lines all passed through the origin. Meanwhile, slopes of lines decreased with increasing of 5OCSA, suggesting that 5OCSA was a reversible XOD inhibitor [34,35].

### 2.3. Molecular Docking of 5OCSA Acid into XOD

Molecular docking is an in silico method by calculating possible interactions between compounds and target protein with structural information from PDB bank. Co-crystallization of XOD with quercetin suggested that the amino acids around molybdenum center, Phe 914, Phe 1009, Arg 880 and Glu 802 are of pivotal importance for the interaction between inhibitors and XOD [33]. In this study, we performed molecular docking of 5OCSA into XOD (PDB: 3NVY, in complex with quercetin), using quercetin as control by Discovery Studio 4.5 software (Figure 2c,d). Similar to quercetin, 5OCSA was well aligned into the molybdenum center surrounding by Leu 648, Lys 771, Ser 876, Arg 880, Phe 914, Phe 1009, Thr 1010, Val 1011, Phe 1013 and Ala 1079, with a binding energy of −8.6 kcal/mol. Dihydroxycyclohexene-1-carboxylic acid ring of 5OCSA were sandwiched between Phe 914 and Phe 1009 and dihydroxyphenyl ring were positioned in the molybdenum center. Notably, 5OCSA forms hydrogen bond with XOD through Lys 771, Ala 1079, Val 1011 and Thr 1010.

### 2.4. Effect of 5OCSA on Body Weight and Organ Coefficients in HUA Mice

**Figure 3:**
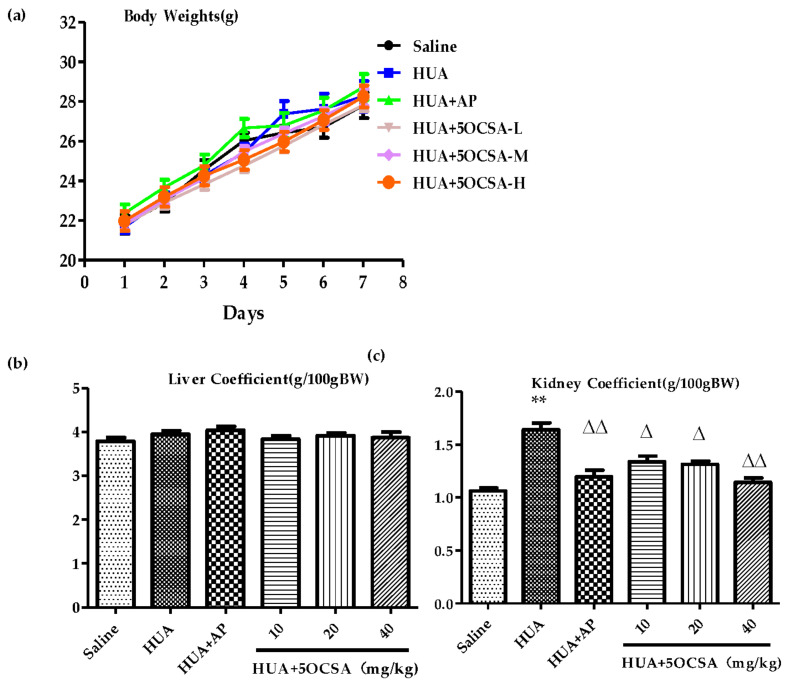
Effects of different doses of 5OCSA on BWs and organ coefficients in HUA mice. (**a**) Changes of BWs in different groups (n = 8 in each group). Coefficients of liver (**b**) and kidney (**c**) were calculated and plotted via GraphPad. ** *p* < 0.01, compared with saline group; ∆ *p* < 0.05; ∆∆ *p* < 0.01, compared with HUA group. 5OCSA-L: low dose of 5-O-caffeoylshikimic acid; 5OCSA-M: medium dose of 5OCSA; 5OCSA-H: high dose of 5OCSA; BWs: bodyweights; HUA: hyperuricemia.

To investigate whether 5OCSA could ameliorate HUA in vivo, we generated a PO/HX-induced HUA mice model as described in 4.4. As shown in Figure 3a, during the 7 days of HUA modeling, the body weights (BWs) increased steadily and there is no statistically differences among groups. Additionally, organ coefficients (liver and kidney) have also been investigated. There are no significant differences in liver coefficients among groups (Figure 3b), while kidney coefficients of HUA mice increased significantly, compared with the normal saline group (Figure 3c, 1.64 g/100g BW vs. 1.06 g/100g BW, *p* < 0.01). Compared with HUA group, different doses of 5OCSA could all significantly reduce the kidney coefficient (all *p* < 0.05), indicating 5OCSA probably has nephroprotective effects in HUA mice. Therefore, no obvious harmful effects in BWs and organ coefficient (liver and kidney) have been observed in HUA mice when treated with 40 mg/kg BWs or below of 5OCSA.

### 2.5. Effect of 5OCSA on sUA, Serum XOD and Hepatic XOD

**Figure 4:**
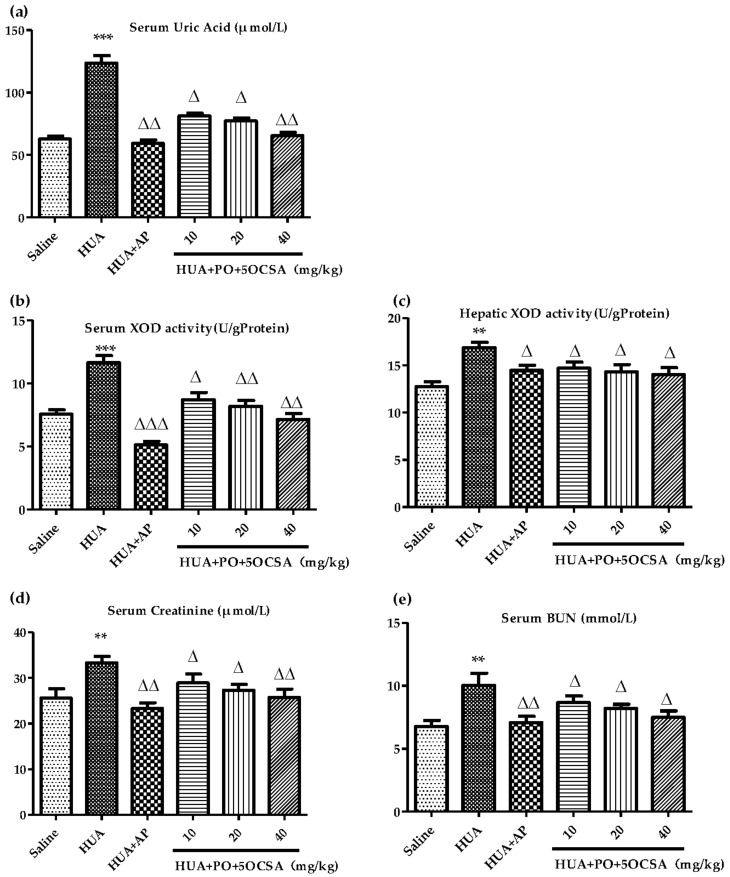
Effects of 5OCSA on sUA, XOD and markers of kidney damage in HUA. (**a**) Serum level of uric acid (μmol/L). (**b**) Serum XOD activity (U/g Protein). (**c**) Hepatic XOD activity (U/g Protein). (**d**) Serum creatinine (μmol/L). (**e**) Serum BUN (mmol/L). ** *p* < 0.01, *** *p* < 0.005, compared with saline group; ∆ *p* < 0.05; ∆∆ *p* < 0.01, ∆∆∆ *p* < 0.005, compared with HUA group. sUA: serum uric acid; XOD: xanthine oxidase; BUN: blood urea nitrogen.

After seven consecutive days of PO treatment, the animals were sacrificed and sUA concentrations were evaluated. In comparison with the saline group (62.70μmol/L), the PO/HX treated HUA group increased significantly to 123.51 μmol/L (*** *p* < 0.005), suggesting the HUA model has been successfully replicated (Figure 4a). Compared with HUA group, three different doses of 5OCSA groups and AP group (HUA + 20 mg/kg AP), have all shown significant reduction in the elevated sUA level (all ∆ *p* < 0.05), suggesting 5OCSA could ameliorate HUA in vivo.

To verify the XOD inhibition effects of 5OCSA in vivo, we evaluated the XOD activity in liver and serum. As shown in Figure 4b,c, after seven consecutive days treatment of activities of PO/HX, serum XOD and hepatic XOD increased by 53.97% and 32.31% respectively compared with saline group (11.64 vs. 7.56 U/g Protein); 16.87 vs. 12.75 U/g Protein, all ** *p* < 0.01). Compared with HUA group, 5OCSA at 10, 20 and 40 mg/kg notably and dose-dependently decreased XOD activities by 25.17%, 29.64% and 38.66%, respectively, in HUA mice liver (8.71, 8.19 and 7.14 vs. 11.64 U/g Protein). Similarly, compared with HUA group, 5OCSA at 10, 20 and 40 mg/kg also dose-dependently decreased XOD activities by 12.80%, 15.18% and 16.89%, respectively, in HUA mice liver (14.71, 14.31 and 14.02 vs. 16.87 U/g Protein). Results mentioned above suggested that 5OCSA could also inhibit XOD in vivo.

### 2.6. OCSA Ameliorates Kidney Injury in PO/HX-Induced HUA Mice

**Figure 5:**
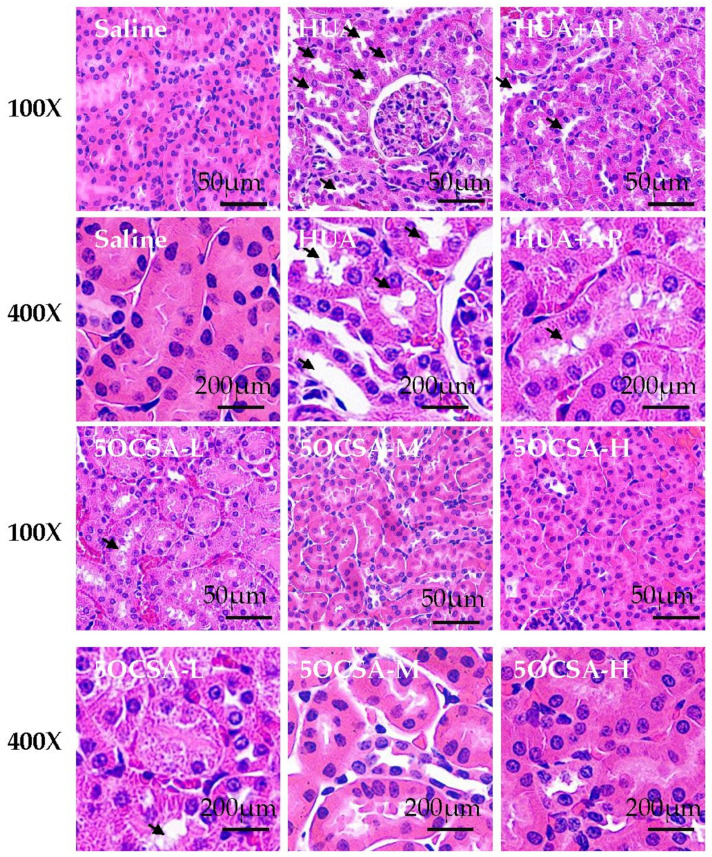
Protective effects of 5OCSA on kidney histomorphometry change caused by PO/HX in mice. The right kidney of mice was fixed, embedded in paraffin, sectioned, and stained by H&E. Arrows indicate the enlarged vacuoles in renal tubular epithelial cells. 100*×*: 100-fold magnification; 400*×*: 400-fold magnification; 5OCSA: 5-*O*-caffeoylshikimic acid; 5 OCSA-L: low dose of 5OCSA; 5OCSA-M: medium dose of 5OCSA; 5OCSA-H: high dose of 5OCSA.

Creatinine (Cr) and Blood urea nitrogen (BUN), two kidney injury indicators, were also examined. As shown in Figure 4d,e, the serum Cr and BUN in HUA group increased by 30.31% and 48.52% as compared to saline group respectively (33.32 vs. 25.57 μmol/L; 10.04 vs. 6.76 mmol/L), suggesting occurrence of kidney injury in HUA mice. Compared to the HUA group, different doses of 5OCSA could significantly reduce Cr level by 13.24% (10mg/kg BWs, 28.91 μmol/L), 18.00% (20 mg/kg BWs, 27.33 μmol/L) and 22.69% (40mg/kg BWs, 25.76 μmol/L) as well as the BUN level by 13.45% (10 mg/kg BWs, 8.69 mmol/L), 18.23% (20 mg/kg BWs, 8.21 mmol/L) and 25.40% (40 mg/kg BWs, 7.49 mmol/L), indicating 5OCSA probably has a protective effect on kidney injury in HUA mice. To examine the structural deterioration in the kidneys of HUA mice, we performed H&E staining. In the saline group, the renal tubulars of mice were intact and smooth. Compared to the saline group, the HUA model group exhibited tubular dilation and vacuole in renal tubular epithelial cells. Compared to HUA group, the levels of renal tubular dilation were significantly reduced after the administration of different doses of 5OCSA (Figure 5).

### 2.7. Effect of 5OCSA on Inflammatory Cytokines of Kidney in HUA Mice

**Figure 6:**
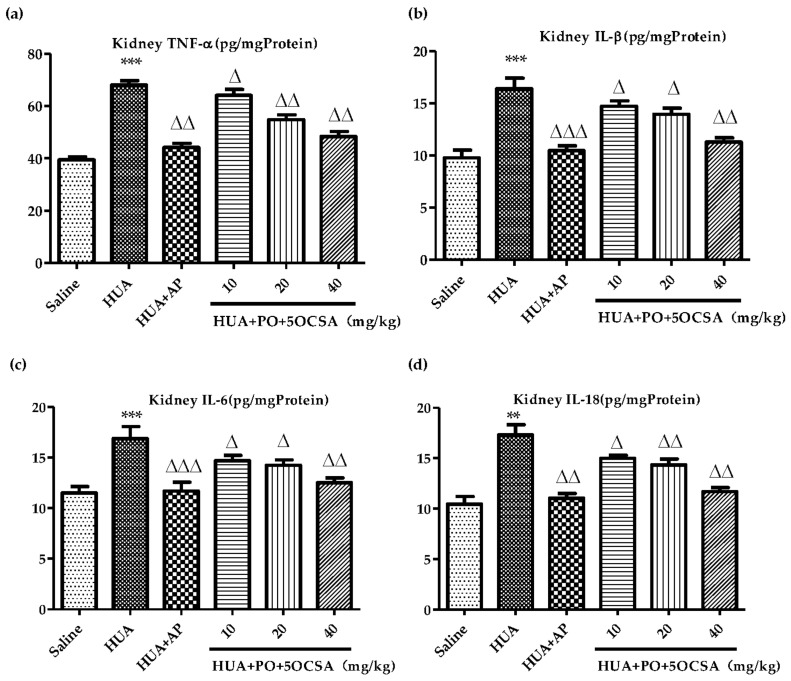
Effects of 5OCSA on kidney inflammation cytokines in HUA mice. (**a**) TNF-α (pg/mg Protein); (**b**) IL-1β (pg/mg Protein); (**c**) IL-6 (pg/mg Protein); (**d**) IL-18 (pg/mg Protein). Data are represented as mean ± SD (n = 8). ** *p* < 0.01, *** *p* < 0.005, compared with saline group; ∆ *p* < 0.05; ∆∆ *p* < 0.01, ∆∆∆ *p* < 0.005, compared with HUA group. TNF-α: tumor necrosis factor-α; IL-1β: interleukin-1β; IL-6: interleukin-6; IL-18: interleukin-18.

Activation of inflammatory cytokines in injury kidney were frequently reported in HUA patients and animal models [36,37,38].To assess the effects of 5OCSA on inflammatory cytokines, we evaluated the changes of inflammatory cytokines TNF-α, IL-1β, IL-6 and IL-18 in kidney by Elisa. As shown in Figure 6, TNF-α, IL-1β, IL-6 and IL-18 in HUA mice increased by 72.44%, 68.13%, 46.89% and 65.67%, respectively, (68.01 vs. 39.44 pg/mg Protein; 16.39 vs. 9.75 pg/mg Protein; 16.89 vs. 11.5 pg/mg Protein; 17.31 vs. 10.45 pg/mg Protein), suggesting that the inflammatory cytokines we examined had been activated by HUA. Compared with the HUA group, different doses of 5OCSA could all significantly and dose-dependently reduce the elevated cytokines (all ∆ *p* < 0.05), suggesting 5OCSA could effectively ameliorate kidney inflammation in HUA.

## 3. Discussion

SGR, as a traditional Chinese medicine with more than 2000 years of history, has long been acclaimed as an effective anti-hyperuricemia agent. Consistently with the traditional use from ancient China, in 2011, water extract of SGR (SGR-WE)was first found to ameliorate HUA in rats and inhibit XOD activity by Guo et al. [39], which inspired researchers to find the bioactive compounds responsible for the anti-HUA effects. Similarly, Hong et al. found that the crude drug of SGR could also ameliorate HUA rats, and interpreted the hypouricemia effects of SGR as being able to reduce ROS by upregulating catalase[28]. In 2013, Xu et al. [29] performed a correlation study by comparing the components of ethanol extract of SGR (SGR-EE) and the serum of HUA rats after administration of SGR-WE. They claimed seven compounds might be related with HUA including glucuronide form of 5OCSA. In 2019, Liang et al.[23] replicated Guo’s work in a chronic HUA/gout mice model and found SGR-WE could ameliorate HUA and gout phenotypes. They claimed nine compounds from SGR-WE were identified by HPLC-DAD-MS/MS. Therefore, SGR extracts should contain compounds promising the development of natural drugs preventing HUA. However, none of the works mentioned above have verified the anti-HUA effects of individual compound isolated. Clinically, the current major drugs for sUA-lowering are still XOD inhibitors including AP, febuxostat and topirastat. However, the undesirable adverse effects limited their clinical application. Therefore, in this study, we aimed to identify natural XOD inhibitors from SGR extract by XOD inhibitory assay-guided separation and verified the XOD inhibitory effects in vitro and in vivo.

Extraction and isolation of compounds from plants are quite time-consuming. In this study, we evaluated the XOD-inhibitory capacity of each fraction achieved before further separations. Through this so-called bio-assay guided separation, the smaller fraction, EAF (EAF 182.4 g < BUF 1584.0 g) was kept and further subjected to a silica gel column. Similarly, sub-fractions CMF2 and CMF5 were further isolated as shown in Figure S1. Finally, we obtained six known compounds according to the physicochemical properties, NMR spectral analysis and comparison with publications. Among them, 5OCSA, together with quercetin and astilbin, were identified as natural XOD inhibitors. Whether there remains new compounds in other fractions, especially for non-XOD inhibitors studies, still requires more investigations in the future.

5OCSA is a bioactive polyphenolic compound isolated from multiple plants or foods, including *Phoenix dactylifera* [40], *Zanthoxylum naranjillo* leaves [41], *Solanum somalense* leaves [42], *Caryota urens* L. [43], Palm oil [44] and SGR [29,32]. Functionally, 5OCSA has complicated bioactivities including anti-thiamine factor [45], anti-oxidant [44],α-glucosidase inhibitory activity [43] and anti-inflammatory [46]. To our knowledge, the function of 5OCSA in HUA still remains unclear. From the XOD inhibitory assay in vitro (Figure 2a), 5OCSA has been identified as an effective XOD inhibitor with IC_50_ of 13.96 μM. The reversibility study (Figure 2b) and molecular docking (Figure 2c) suggested that 5OCSA might be a reversible XOD inhibitor by binding with the molybdopterin domain, the active site of XOD [47]. According to the co-crystallization of XOD with quercetin, amino acids around molybdenum center, Phe 914, Phe 1009, Arg 880, and Glu 802 are of pivotal importance for the interaction between inhibitors and XOD [33]. Similar to quercetin, the dihydroxycyclohexene-1-carboxylic acid ring of 5OCSA was sandwiched between Phe 914 and Phe 1009, and 5OCSA fits the active pocket of molybdopterin domain well surrounded by Leu 648, Lys 771, Ser 876, Arg 880, Phe 914, Phe 1009, Thr 1010, Val 1011, Phe 1013 and Ala 1079. The in vivo inhibition efficiencies on XOD in the liver and serum are also impressive. Collectively, from the in vitro, in silico and in vivo evidence, 5OCSA is a promising effective natural XOD inhibitor.

The nephroprotective role of 5OCSA in HUA mice was also examined. As a major comorbidity combined with HUA clinically, kidney damage along with reduced clearance of creatinine and urea also existed in HUA mice. In our study, kidney coefficients (Figure 3c), serum levels of Cr (Figure 4d) and BUN (Figure 4e) in HUA group increased by 54.72%, 30.31% and 48.52% compared with a blank control group, suggesting the occurrence of kidney injury in HUA, which could be dose-dependently compromised by 5OCSA. Notably, the high dose of 5OCSA (40 mg/BWs) could reduce the elevated kidney coefficients, serum levels of Cr and BUN in were reduced by 29.88%, 22.69% and 34.05%, suggesting the nephroprotective effects of 5OCSA in HUA. Moreover, histopathological analysis by H&E staining (Figure 5) further validated the kidney injury in HUA and nephroprotective effects of 5OCSA. Another indicator of kidney injury in HUA examined in this study are inflammatory cytokines. Compared with saline group, TNF-α, IL-1β, IL-6 and IL-18 in kidney tissue of HUA mice, have been increased by 72.44%, 68.13%, 46.89% and 65.67%, respectively (Figure 6). Compared with the HUA group, 5OCSA could dose-dependently compromise the elevated cytokines in HUA mice, and high dose group (40 mg/BWs) could reduce the elevated TNF-α, IL-1β, IL-6 and IL-18 by 19.60%, 31.28%, 25.87%, and 32.47%, respectively. Taken together, our study suggested 5OCSA played nephroprotective role in HUA mice. Considering the dual roles of pro-inflammatory cytokines in HUA kidneys, indicator and inducer, the mechanism of 5OCSA interaction with cytokines required further investigation. Meanwhile, whether nephroprotective effects of 5OCSA could be realized through XOD inhibition independent mechanism should also be addressed in future study.

Natural products have long been considered as safe resources for drug development with few side effects [48,49]. As a strong XOD inhibitor used clinically, AP performed well in reducing sUA in PO/HX induced HUA mice (Figure 4a) as expected. Strikingly, as shown in Figure 5, the histological defect of kidney was not well restored by AP which might be interpreted as side effects of AP on kidney. In this study, we examined the BWs and organ coefficients for the toxicity or side effects study of 5OCSA. As shown in Figure 3b,c, liver coefficients are stable in various groups, but kidney coefficients are elevated in the HUA group. However, different doses of 5OCSA could reduce the elevation to a certain extent, indicating 5OCSA can antagonize the effect of PO/HX on kidney. Meanwhile, BWs of each groups group stably without statistical difference among groups. Taken together, 5OCSA has little toxicity for the mice.

## 4. Materials and Methods

### 4.1. Material

SGR was obtained from Tongrentang Pharmacy (Changchun, China) and a voucher sample (#SGR-ZD-2020073106) was reserved in College of Pharmacy, Changchun University of Chinese Medicine. Isolation columns including macropore adsorptive resin D101 (A832686), column-layer chromatographic silica gel (C820912), Sephadex LH-20 (S822637), sodium carboxymethylcellulose (CMC-Na, C804625) and hypoxanthine (G1725018) were all obtained from Macklin (Shanghai, China). EtOH, EtOAc, CH_2_Cl_2_, CHCl_3_ and MeOH were purchased from Aladdin (Shanghai, China). XOD (X4500-25UN), AP (8003-25G), xanthine (X7375-25G), potassium oxonate (PO, 156124), PBS (806552-500ML), NaOH (S8045-500G), KH_2_PO4 (P0662-500G) and DMSO (D8418-100ML) used for detection of enzyme activity were all purchased from Merck (Chengdu, China).

### 4.2. Separation

6.0 kg of dried SGR was crushed and extracted by 100 L 70% EtOH three times for 4 h at 50 °C. Removing EtOH from the extract under reduced pressure and partitioned with n-Hexane till the water layer is colorless. Evaporated the remaining water and achieved 3.32 kg of crude ethanol extracts (CEE). Dissolved 3.0 kg CEE with 30% EtOH and subjected to macropore adsorptive resin D101. Eluted the resin with distilled water and 70% ethanol successively and partitioned with n-BuOH and EtOAc successively. 182.4 g of EtOAc soluble fraction (EAF) and 1584.0 g n-BuOH soluble fraction (BUF) were obtained. The EAF fraction (150 g) was separated by silica column and eluted by gradient CHCl_3_-MeOH solutions successively (30:1, 25:1, 20:1, 15:1, 10:1, 5:1 and 0:1 *v*/*v*). The seven fractions were collected and the solvent was recovered under reduced pressure, obtaining CMF1 (4.0 g, 30:1), CMF2 (8.4 g, 25:1), CMF 3 (12.7 g, 20:1), CMF4 (11.5 g, 15:1), CMF5 (9.5 g, 10:1), CM6 (6.4 g, 5:1) and CMF7 (2.1 g, 0:1), respectively.

CMF2 was separated by Sephadex LH-20 and two fractions were obtained. CMF2.1 and 2.2. CMF2.1 was recrystallized in MeOH and compound **3** was obtained (28.4 mg). CMF2.2 was added to silica gel column chromatography and the obtained CMF2.2.1 and CMF2.2.2 was eluted with CH_2_Cl_2_-MeOH (15:1 and 1:1), respectively. CMF2.2.1 was then subjected to semi-preparative HPLC (UltiMate 3000, Thermofisher, Germering, Germany) (MeOH-H_2_O, 25:75) and compound **4** was obtained (159.8 mg); CMF2.2.2 was recrystallized in MeOH and compound **5** was obtained (133.1 mg). CMF5 was recrystallized in MeOH and obtained compound **1** (114.6 mg). The remaining solutions were added to silica gel column chromatography again and the obtained CMF5.1-5.3 was eluted with CH2Cl2-MeOH (12:1, 10:1 and 8:1), respectively. CMF5.1 was subjected to semi-preparative HPLC (MeOH-H_2_O, 50:50) and the obtained compound **2** (203.7 mg). CMF5.3 was recrystallized (methanol) to obtain compound **6** (463.5 mg).

### 4.3. XOD Inhibitory Assay and Reversibility Study

XOD enzyme activity was measured by the spectrophotometry (INESA/752N, Shanghai Yidian Company, Shanghai, China) of 295 nm indicating the mount of uric acid [50]. The reaction mixture is 200 μL, including 40 μL XOD (0.05 U, final concentration 250 U/L), 50 μL of 1 mM xanthine, 10 μL of sample solution dissolved in DMSO, and 100 μL of 2 mM phosphate buffer (pH 7.5). For the control group and blank group, 10 μL DMSO and 40 μL phosphate buffer replaced 10 μL test sample and 40 μL XOD respectively. Each reaction was examined in triplicate. Different concentrations of extractions (CEE, EAF, BUF and CMFs (1–7)) for XOD inhibition assay were all tested. To obtain the IC_50_ values, compounds isolated from fractions CMF2 and CMF5 (1 to 6) were evaluated in five different concentrations and plotted via Graphpad. All reactions were examined in triplicate. Inhibition rates of XOD was calculated as (Absorbance control − Absorbance samples)/(Absorbance control − Absorbance blank) × 100%. The inhibition reversibility study of 5OCSA on XOD was performed by using 5 different concentration of compounds (0.00, 3.75, 7.50, 15.00, and 30.00 μM) and 5 different concentrations of XOD (50.0, 100.0, 150.0, 200.0 and 250.0 U/L). Plots of OD_295_/min vs. concentration of XOD were generated by Graphpad Prism 5 (version 5.01, GraphPad Company, San Diego, CA, USA).

### 4.4. Animals Groupings and Experiment Procedure

Kunming mice (License # SCXK (Liao) 2020–0001) were all obtained from Changsheng Biotech (Benxi, China) and maintained under 12 h day/night cycle and 21 ± 2 °C temperature. 48 mice (six weeks old, 21.0 ± 2.0 g) were randomly divided into 6 groups, saline group (15 mL/kg BWs saline via intraperitoneal injection (i.p.); 20 mL/kg BWs 0.5% CMC-Na via intragastric (i.g.) gavage×7 days), HUA group (300 mg/kg/day PO via i.p. injection×7 days, HX 600 mg/kg/day via i.g. gavages×7 days), HUA+AP or positive control group (HUA; 20 mg/kg/day AP via i.g. gavages×7 days), HUA+5OCSA-L or low dose 5OCSA group (HUA; 10 mg/kg/day 5OCSA via i.g. gavages×7 days), HUA+5OCSA-M or medium dose 5OCSA group (HUA; 20 mg/kg/day 5OCSA via i.g. gavages×7 days) and HUA+5OCSA-H or high dose 5OCSA group (HUA; 40 mg/kg/day 5OCSA via i.g. gavages×7 days). PO was dissolved into 25 mg/mL by sterile saline. HX and 5OCSA were dissolved into 60mg/mL and 40 mg/mL respectively by 0.5% CMC-Na. At day 7, blood was collected 1 h after drug administration and kept still in room temperature for 1 h. Centrifuged at 5000× *g* and 25 °C for 10 min and collect the supernatant. Serum uric acid levels were examined by uric acid test kit (C012-2-1, Nanjing Jiancheng) according to the protocol. Experiments were permitted by the ethics committee of College of Pharmacy, Changchun University of Chinese Medicine.

### 4.5. HE Staining and Kidney Histomorphometry

Fix the right kidney in 4% para-formaldehyde solution (in PBS) for 3 days at room temperature. Embedded the samples with paraffin and sectioned the specimen in 5 μm slices. Stained with hematoxylin and eosin (HE) and observed under light microscope (Osteoplan II; Carl Zeiss, NY, USA).

### 4.6. Measurement of Uric Acid Concentration and XOD Activity

Concentrations of serum uric acid were determined by a standard kit from Nanjing Jiancheng (C012-1-1, Nanjing, China). Considering the low solubility of uric acid, the serum samples were diluted 10-fold at 50 °C. 200 μL test samples or standard solution (50 mg/L or 297.4 μM uric acid) were mixed with 2.0 mL Buffer P gently and let stand for 10 min. The samples were then centrifuged at 3000× *g* for 5 min and collected the supernatant. The supernatant was mixedwith Buffer A and Buffer B gently and let stand for 10 min at room temperature. The absorption value in 690 nm was examined by spectrophotometer. XOD activity from serum and liver samples were examined by spectrophotometric method with a XOD assay kit obtained from Nanjing Jiancheng (A002-1-1, Nanjing, China) [51]. Each test has been performed in triplicate.

### 4.7. Molecular Docking

3NVY [33] is the entry code of bovine XOD co-crystallization with quercetin retrieved from RCSB PDB, with a resolution of 2.0 Å. In this study, discovery studio 4.5 (BIOVIA, San Diego, CA, USA) was used for the docking of 5-*O*-caffeoylshikimic acid and quercetin into 3NVY. For the preparation of docking, we removed all water molecules and added charges and hydrogen to 3NVY [51]. The compounds were docked using the CDOCKER protocol with default settings.

## 5. Conclusions

Considering the pharmacological effects of SGR extracts in reducing uric acid in HUA, we performed a XOD inhibitory assay-coupled separation of fractions and compounds from SGR. Our results revealed that CMF2 and CMF5 derived from EAF showed obvious XOD inhibitory potency, with inhibitory rate of 39.82% and 52.03% at 200 μg/mL, respectively. Through further isolation, we obtained three XOD inhibitors, 5OCSA with IC_50_ of 13.96 μM, as well as two known XOD inhibitors, quercetin (#3),and astilbin (#6) from CMF2 and CMF5. Furthermore, 5OCSA has been successfully docked into the active pocket of XOD (PDB #3NVY), suggesting the existence of physical interaction between XOD and 5OCSA. To evaluate the in vivo effect of 5OCSA on HUA, we generated a PO/HX induced HUA model. 5OCSA could inhibit both hepatic and serum XOD in vivo, together with reducing the sUA level. Moreover, different doses of 5OCSA could all effectively and dose-dependently compromise the elevated serum level of BUN and Cr and the histological defect and activation of cytokines in kidneys, suggesting the nephroprotective effects of 5OCSA on kidney injury in HUA. Collectively, our results suggested that 5OCSA may be developed into a safe and effective XOD inhibitor based on in vitro, in silico and in vivo evidence.

## Data Availability

Date of the compounds is available from the authors.

## Supplementary Materials

The following are available online. Figure S1: Detailed procedure of compounds isolated from CMF2 and CMF5; Figure S2: Structure analysis of the total 6 compounds.

## Sample Availability

Samples of the compounds are available from the authors.

## References

[1] Zhang S., Wang Y., Cheng J., Huangfu N., Zhao R., Xu Z., Zhang F., Zheng W., Zhang D. (2019). Hyperuricemia and Cardiovascular Disease. Curr. Pharm. Des..

[2] Liu S. (1995). Danxi Xinfa and Zhu Danxi’s correlated works. Zhonghua Yi Shi Za Zhi.

[3] Shahin L., Patel K.M., Heydari M.K., Kesselman M.M. (2021). Hyperuricemia and Cardiovascular Risk. Cureus.

[4] Zhou M., Yang N., Xing X., Chang D., Li J., Deng J., Chen Y., Hu C., Zhang R., Lu X. (2021). Obesity interacts with hyperuricemia on the severity of non-alcoholic fatty liver disease. BMC Gastroenterol..

[5] Arersa K.K., Wondimnew T., Welde M., Husen T.M. (2020). Prevalence and Determinants of Hyperuricemia in Type 2 Diabetes Mellitus Patients Attending Jimma Medical Center, Southwestern Ethiopia, 2019. Diabetes Metab. Syndr. Obes. Targets Ther..

[6] Stewart D.J., Langlois V., Noone D. (2019). Hyperuricemia and Hypertension: Links and Risks. Integr. Blood Press. Control.

[7] Toda A., Ishizaka Y., Tani M., Yamakado M. (2014). Hyperuricemia is a significant risk factor for the onset of chronic kidney disease. Nephron. Clin. Pract..

[8] Major T.J., Dalbeth N., Stahl E.A., Merriman T.R. (2018). An update on the genetics of hyperuricaemia and gout. Nat. Rev.. Rheumatol..

[9] Álvarez-Lario B., Alonso-Valdivielso J.L. (2014). Hyperuricemia and gout; the role of diet. Nutr. Hosp..

[10] Li R., Yu K., Li C. (2018). Dietary factors and risk of gout and hyperuricemia: A meta-analysis and systematic review. Asia Pac. J. Clin. Nutr..

[11] Ben Salem C., Slim R., Fathallah N., Hmouda H. (2017). Drug-induced hyperuricaemia and gout. Rheumatology.

[12] Dalbeth N., Gosling A.L., Gaffo A., Abhishek A. (2021). Gout. Lancet.

[13] Huang J., Ma Z.F., Zhang Y., Wan Z., Li Y., Zhou H., Chu A., Lee Y.Y. (2020). Geographical distribution of hyperuricemia in mainland China: A comprehensive systematic review and meta-analysis. Glob. Health Res. Policy.

[14] Abhishek A., Roddy E., Doherty M. (2017). Gout—A guide for the general and acute physicians. Clin. Med..

[15] Hainer B.L., Matheson E., Wilkes R.T. (2014). Diagnosis, treatment, and prevention of gout. Am. Fam. Physician.

[16] Mandal A.K., Mount D.B. (2015). The molecular physiology of uric acid homeostasis. Annu. Rev. Physiol..

[17] Bredemeier M., Lopes L.M., Eisenreich M.A., Hickmann S., Bongiorno G.K., d’Avila R., Morsch A.L.B., da Silva Stein F., Campos G.G.D. (2018). Xanthine oxidase inhibitors for prevention of cardiovascular events: A systematic review and meta-analysis of randomized controlled trials. BMC Cardiovasc. Disord..

[18] Klinenberg J.R. (1965). The effectiveness of allopurinol in the treatment of gout. Arthritis Rheum..

[19] Bardin T., Richette P. (2019). The role of febuxostat in gout. Curr. Opin. Rheumatol..

[20] Khalil N.Y., AlRabiah H.K., Al Rashoud S.S., Bari A., Wani T.A. (2019). Topiramate: Comprehensive profile. Profiles Drug Subst. Excip. Relat. Methodol..

[21] Jordan A., Gresser U. (2018). Side Effects and Interactions of the Xanthine Oxidase Inhibitor Febuxostat. Pharmaceuticals.

[22] Mehmood A., Ishaq M., Zhao L., Safdar B., Rehman A.U., Munir M., Raza A., Nadeem M., Iqbal W., Wang C. (2019). Natural compounds with xanthine oxidase inhibitory activity: A review. Chem. Biol. Drug Des..

[23] Liang G., Nie Y., Chang Y., Zeng S., Liang C., Zheng X., Xiao D., Zhan S., Zheng Q. (2019). Protective effects of Rhizoma smilacis glabrae extracts on potassium oxonate- and monosodium urate-induced hyperuricemia and gout in mice. Phytomedicine: Int. J. Phytother. Phytopharm..

[24] Qiu R., Shen R., Lin D., Chen Y., Ye H. (2008). Treatment of 60 cases of gouty arthritis with modified Simiao Tang. J. Tradit. Chin. Med. = Chung I Tsa Chih Ying Wen Pan.

[25] Han J., Shi G., Li W., Wang S., Bai J., Sun X., Xie Y., Sui F., Chen F., Jiang D. (2021). Zisheng Shenqi Decoction Ameliorates Monosodium Urate-Mediated Gouty Arthritis in Rats via Promotion of Autophagy through the AMPK/mTOR Signaling Pathway. Evid.-Based Complementary Altern. Med. Ecam.

[26] Zhang X.Y., Cheng J., Zhao P., Chen K.L., Li J. (2019). Screening the Best Compatibility of Selaginella moellendorffii Prescription on Hyperuricemia and Gouty Arthritis and Its Mechanism. Evid.-Based Complementary Altern. Med. Ecam.

[27] Chi X., Zhang H., Zhang S., Ma K. (2020). Chinese herbal medicine for gout: A review of the clinical evidence and pharmacological mechanisms. Chin. Med..

[28] Hong Q., Yu S., Mei Y., Lv Y., Chen D., Wang Y., Geng W., Wu D., Cai G., Chen X. (2014). Smilacis Glabrae Rhizoma reduces oxidative stress caused by hyperuricemia via upregulation of catalase. Evid.-Based Complementary Altern. Med. Ecam.

[29] Xu W.A., Yin L., Pan H.Y., Shi L., Xu L., Zhang X., Duan J.A. (2013). Study on the correlation between constituents detected in serum from Rhizoma Smilacis Glabrae and the reduction of uric acid levels in hyperuricemia. J. Ethnopharmacol..

[30] Guo W., Dong H., Wang D., Yang B., Wang X. (2018). Separation of Seven Polyphenols from the Rhizome of Smilax glabra by Offline Two Dimension Recycling HSCCC with Extrusion Mode. Molecules.

[31] Chen T., Li J., Cao J., Xu Q., Komatsu K., Namba T. (1999). A new flavanone isolated from rhizoma smilacis glabrae and the structural requirements of its derivatives for preventing immunological hepatocyte damage. Planta Med..

[32] Cheng S., Ma Y., Peng C., Lu J., Huang H., Shu J. (2021). Chemical Constituents from the Ethyl Acetate Effective Parts of Smilacis Glabrae Rhizoma. J. Chin. Med. Mater..

[33] Cao H., Pauff J.M., Hille R. (2014). X-ray crystal structure of a xanthine oxidase complex with the flavonoid inhibitor quercetin. J. Nat. Prod..

[34] Zhang C., Wang R., Zhang G., Gong D. (2018). Mechanistic insights into the inhibition of quercetin on xanthine oxidase. Int. J. Biol. Macromol..

[35] Wang Y., Curtis-Long M.J., Lee B.W., Yuk H.J., Kim D.W., Tan X.F., Park K.H. (2014). Inhibition of tyrosinase activity by polyphenol compounds from Flemingia philippinensis roots. Bioorg. Med. Chem..

[36] Joosten L.A.B., Crişan T.O., Bjornstad P., Johnson R.J. (2020). Asymptomatic hyperuricaemia: A silent activator of the innate immune system. Nat. Rev. Rheumatol..

[37] Zheng Y., Guan H., Zhou X., Xu Y., Fu C., Xiao J., Ye Z. (2020). The association of renal tubular inflammatory and injury markers with uric acid excretion in chronic kidney disease patients. Int. Urol. Nephrol..

[38] Ruggiero C., Cherubini A., Ble A., Bos A.J., Maggio M., Dixit V.D., Lauretani F., Bandinelli S., Senin U., Ferrucci L. (2006). Uric acid and inflammatory markers. Eur. Heart J..

[39] Guo S.Y., Zhang W., Zhang Y., Du X.Y. (2011). Effect of Smilax glabra Water-extracts on Serum Uric Acid, Triglyceride and Cholesterol in Hyperuricemia Rats. China Pharm..

[40] Farag M.A., Handoussa H., Fekry M.I., Wessjohann L.A. (2016). Metabolite profiling in 18 Saudi date palm fruit cultivars and their antioxidant potential via UPLC-qTOF-MS and multivariate data analyses. Food Funct..

[41] Braguine C.G., Costa E.S., Magalhães L.G., Rodrigues V., da Silva Filho A.A., Bastos J.K., Silva M.L., Cunha W.R., Januário A.H., Pauletti P.M. (2009). Schistosomicidal evaluation of Zanthoxylum naranjillo and its isolated compounds against Schistosoma mansoni adult worms. Z. Fur Naturforschung. C J. Biosci..

[42] Chideh S., Pilard S., Attoumbré J., Saguez R., Hassan-Abdallah A., Cailleu D., Wadouachi A., Baltora-Rosset S. (2014). 5-O-caffeoylshikimic acid from Solanum somalense leaves: Advantage of centrifugal partition chromatography over conventional column chromatography. J. Sep. Sci..

[43] Ferreres F., Andrade C., Gomes N.G.M., Andrade P.B., Gil-Izquierdo A., Pereira D.M., Suksungworn R., Duangsrisai S., Videira R.A., Valentão P. (2021). Valorisation of kitul, an overlooked food plant: Phenolic profiling of fruits and inflorescences and assessment of their effects on diabetes-related targets. Food Chem..

[44] Sambanthamurthi R., Tan Y., Sundram K., Abeywardena M., Sambandan T.G., Rha C., Sinskey A.J., Subramaniam K., Leow S.S., Hayes K.C. (2011). Oil palm vegetation liquor: A new source of phenolic bioactives. Br. J. Nutr..

[45] Fukuoka M. (1982). Chemical and toxicological studies on bracken fern, Pteridium aquilinum var. latiusculum. VI. Isolation of 5-O-caffeoylshikimic acid as an antithiamine factor. Chem. Pharm. Bull..

[46] Lu C.L., Zhu W., Wang M., Xu X.J., Lu C.J. (2014). Antioxidant and Anti-Inflammatory Activities of Phenolic-Enriched Extracts of Smilax glabra. Evid.-Based Complementary Altern. Med. Ecam.

[47] Okamoto K., Kusano T., Nishino T. (2013). Chemical nature and reaction mechanisms of the molybdenum cofactor of xanthine oxidoreductase. Curr. Pharm. Des..

[48] Veeresham C. (2012). Natural products derived from plants as a source of drugs. J. Adv. Pharm. Technol. Res..

[49] Thomford N.E., Senthebane D.A., Rowe A., Munro D., Seele P., Maroyi A. (2018). Natural Products for Drug Discovery in the 21st Century: Innovations for Novel Drug Discovery. Int. J. Mol. Sci..

[50] Lin S., Zhang G., Liao Y., Pan J., Gong D. (2015). Dietary Flavonoids as Xanthine Oxidase Inhibitors: Structure-Affinity and Structure-Activity Relationships. J. Agric. Food Chem..

[51] Yang T.H., Yan D.X., Huang X.Y., Hou B., Ma Y.B., Peng H., Zhang X.M., Chen J.J., Geng C.A. (2019). Termipaniculatones A-F, chalcone-flavonone heterodimers from Terminthia paniculata, and their protective effects on hyperuricemia and acute gouty arthritis. Phytochemistry.

