# Supplementary material for: Natural Xanthine Oxidase Inhibitor 5-O-Caffeoylshikimic Acid Ameliorates Kidney Injury Caused by Hyperuricemia in Mice
Source: Molecules. 2021 Dec 1;26(23):7307. doi: 10.3390/molecules26237307 (PMC8659034; doi:10.3390/molecules26237307)
Supplement: Supplementary file 1 [file molecules-26-07307-s001.zip › molecules-1476187-supplementary.pdf]

molecules

MDPI

Supplementary Data

Article

Natural Xanthine Oxidase Inhibitor 5-O-Caffeoylshikimic Acid Ameliorates Acute Kidney Injury Caused by Hyperuricemia in Mice

Dong Zhang[1†], Mojiao Zhao[2†], Yumei Li[1],Dafang Zhang[2], Yong Yang[3*] and Lijing Li[1*]

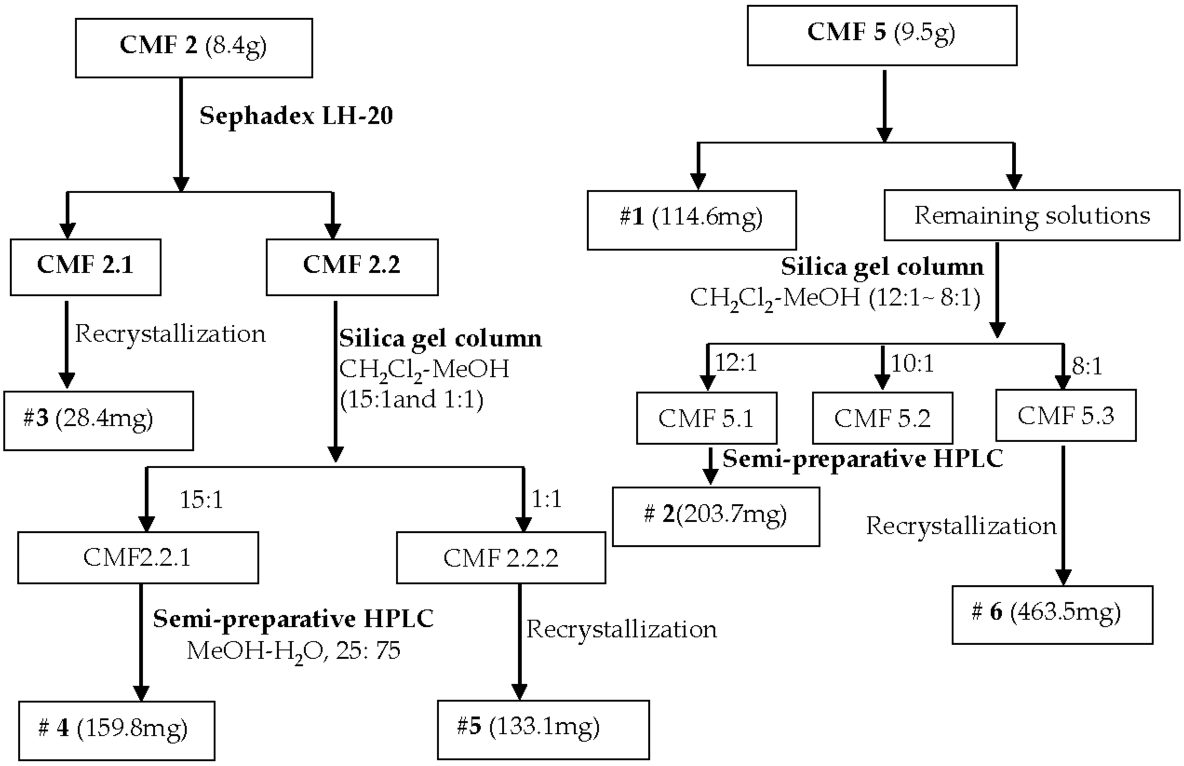

Figure S1: Detailed procedure of compounds isolated from CMF2 and CMF5.

Figure S2: Structure analysis of the total 6 compounds

Compound **1** (*5-O-caffeoylshikimic acid*): Pale brown powder (MeOH). ESI‑MS m/z:335.1 $[M-H]^-$; Molecular formula is $C_{16}H_{16}O_8$.^{1}H NMR (500 MHz, CD_3OD)δ: 7.52 (1H, d, *J* = 16.4 Hz, H‑9), 7.05 (1H, d, *J* = 2.3 Hz, H-2′), 6.98 (1H ,dd, *J* = 8.5, 2.4 Hz, H‑6′), 6.79 (1H, d, *J* = 8.6 Hz, H-5′), 6.72 (1H,m,H-2), 6.20 (1H ,d, *J* = 16.2 Hz, H‑8), 5.28 (1H, m, H-5),4.43 (1H, m, H‑3), 4.14 (1H ,m, H‑4), 2.89 (2H ,m, H‑6).

Compound **2** (*engeletin*): White crystalline powder (MeOH). ESI-MS m/z:433.1[M-H]$^-$; Molecular formula is $C_{21}H_{22}O_{10}$.^{1}H NMR (500 MHz, CD_3OD) δ(ppm): 7.24 (2H, d, *J* = 8.8 Hz, H-2',6'), 6.79 (2H,d,*J* = 8.8 Hz, H-3',5'), 5.94 (1H, s, H-8), 5.89 (1H, s, H-6), 5.30 (1H, d, *J* = 9.6 Hz, H-2),4.68 (1H, d, *J* = 3.8 Hz, H-3), 3.86(1H, d, *J* = 5.5 Hz, H-1''), 3.72 (1H, dt, *J* = 10.4, 5.2 Hz, H-3''), 3.65 (1H, dd, *J* = 13.5, 5.4 Hz, H-2''), 1.24 (3H, d, *J* = 6.5 Hz, H-6'').

Compound **3** (*quercetin*): Yellow amorphous powder (MeOH).ESI-MS m/z: 301.0 [M-H]$^-$; Molecular formula is $C_{15}H_{10}O_7$. ^{1}H NMR (500 MHz, CD_3OD δ: 9.47 (1H, s, 3-OH), 7.68 (1H, s, 3′-OH), 7.45 (1H, d, J = 2.1 Hz, H-2′), 7.34 (1H, dd, J = 8.5, 2.1 Hz, H-6′), 6.79 (1H, d, *J* = 8.8 Hz, H-5′), 6.29 (1H, d, J = 2.0 Hz, H-8), 6.20 (1H, d, J = 2.0 Hz, H-6).

Compound **4** (*shikimic acid ethyl ester*): Yellow oily liquid. ESI-MS m/z:201.1 [M-H]$^-$; Molecular formula is $C_9H_{14}O_5$. ^{1}H NMR (500 MHz, CD_3OD) δ: 6.65 (1H, d, *J* = 7.6 Hz, H-2), 4.54 (1H, d, *J* = 6.4 Hz, 3-OH), 4.46 (1H, m, H-3), 4.22 (2H, q, *J* = 7.2 Hz, H-8), 4.05 (1H, m, H-5), 3.83 (1H, d, *J* = 6.3 Hz, 5-OH), 3.76 (1H, m, H-4), 3.58 (1H, d, *J* = 6.7 Hz, 4-OH), 2.55 (2H, m, H-6), 1.24 (3H, d, *J* = 7.5 Hz, H-9).

Compound **5** (*taxifolin*): White amorphous powder (MeOH). ESI-MS m/z:303.1 [M-H]$^-$; Molecular formula is $C_{15}H_{12}O_7$. ^{1}H NMR (500 MHz, CD_3OD) δ: 6.86 (1H, m, H-2'), 6.79(1H, m, H-6') , 6.75(1H, m, H-5'), 5.93 (1H, d, *J* = 2.0 Hz, H-8), 5.89 (1H, d, J = 2.0 Hz, H-6), 5.07 (1H, d, *J* = 7.3 Hz, H-2), 4.67(.1H, m, H-3).

Compound **6** (*astilbin*): White powder (MeOH). ESI-MS m/z: 449.3 [M-H]$^-$; Molecular formula is $C_{21}H_{22}O_{11}$. ^{1}H NMR (500 MHz, CD3OD) δ: 6.84 (1H, s, H-2'), 6.74 (2H,m, H-5', 6'), 5.89 (2H, dd, *J* = 9.2, 1.9 Hz, H-6, 8), 5.41 (1H, d, *J* = 8.5 Hz, H-2), 4.98 (1H, d, J = 6.3 Hz, H-3), 4.66 (1H, m, H-1''), 3.81 (1H, m, H-5''), 3.47 (1H, m, H-4''), 2.21 (1H, d, J = 6.5 Hz, H-3''), 1.25 (1H, d, J = 6.5 Hz, H-6'').
